# Parental risk factors for congenital diaphragmatic hernia – a large German case-control study

**DOI:** 10.1186/s12887-021-02748-3

**Published:** 2021-06-14

**Authors:** Felicitas Schulz, Ekkehart Jenetzky, Nadine Zwink, Charlotte Bendixen, Florian Kipfmueller, Neysan Rafat, Andreas Heydweiller, Lucas Wessel, Heiko Reutter, Andreas Mueller, Thomas Schaible

**Affiliations:** 1 Department of Neonatology and Pediatric Intensive Care, Children’s Hospital, University of Bonn, Bonn, Germany; 2 Department of Hematology, Oncology and Clinical Immunology, University Hospital Düsseldorf, Düsseldorf, Germany; 3 Department of Child and Adolescent Psychiatry, University Medical Center of the Johannes Gutenberg University Mainz, Mainz, Germany; 4 Faculty of Health, School of Medicine, University of Witten/Herdecke, Witten, Germany; 5 Department of Surgery, University Hospital Bonn, Bonn, Germany; 6 Department of Neonatology, University Children’s Hospital Mannheim, University of Heidelberg, Mannheim, Germany; 7 Department of Pediatric Surgery, University Hospital Bonn, Bonn, Germany; 8 Department of Pediatric Surgery, University Hospital Mannheim, Mannheim, Germany; 9 Institute of Human Genetics, University Hospital Bonn, Bonn, Germany

**Keywords:** Birth defect, CDH, Congenital diaphragmatic hernia, Environmental risk factor

## Abstract

**Background:**

Evidence for periconceptional or prenatal environmental risk factors for the development of congenital diaphragmatic hernia (CDH) is still scarce. Here, in a case-control study we investigated potential environmental risk factors in 199 CDH patients compared to 597 healthy control newborns.

**Methods:**

The following data was collected: time of conception and birth, maternal BMI, parental risk factors such as smoking, alcohol or drug intake, use of hairspray, contact to animals and parental chronic diseases. CDH patients were born between 2001 and 2019, all healthy control newborns were born in 2011. Patients and control newborns were matched in the ratio of three to one.

**Results:**

Presence of CDH was significantly associated with maternal periconceptional alcohol intake (odds ratio = 1.639, 95% confidence interval 1.101–2.440, *p* = 0.015) and maternal periconceptional use of hairspray (odds ratio = 2.072, 95% confidence interval 1.330–3.229, *p* = 0.001).

**Conclusion:**

Our study suggests an association between CDH and periconceptional maternal alcohol intake and periconceptional maternal use of hairspray. Besides the identification of novel and confirmation of previously described parental risk factors, our study underlines the multifactorial background of isolated CDH.

## Background

Congenital diaphragmatic hernia (CDH) represents a severe birth-defect with an estimated birth prevalence of 1 in 2500 newborns [1]. It is characterized by a defect in the embryonic development of the diaphragm which results in an incomplete separation of the abdominal cavity and the thoracic cavity. This incomplete separation leaves to variable degrees stomach, intestine, liver or spleen displaced in the thoracic cavity compromising pulmonary development with concomitant pulmonary hypoplasia and secondary pulmonary hypertension at birth [2]. Normally, the diaphragm develops during the fourth week of gestation and closes until twelfth week [3]. Different types of CDH are classified according to their location. The most common type is the posterolateral Bochdalek hernia, a defect in the lumbocostal trigonum with herniation mostly resulting left-sided [4]. Current knowledge on the underlying cause for CDH is still scarce. Genetic factors might play an important role since about 20% occur as part of a genetic defect or syndrome [5]. In the majority of cases CDH presents as an isolated defect or as one of many malformations without any genetic abnormality detected [6]. The most common co-occuring malformations compromise the cardiovascular, genitourinary, gastrointestinal or the central nervous system [7]. Besides genetic factors the following environmental risk factors have been previously associated with the development of CDH: maternal body mass index (BMI) [8], maternal age [9, 10], pre-gestational diabetes [11], maternal pre-gestational hypertension [12], pre-conceptional diet, especially riboflavin supplementation [13].

In the present study, we hypothesized that periconceptional and prenatal environmental factors have a greater impact on the development of CDH than previously assumed [14]. Here we compared the impact of parental periconceptional smoking and alcohol intake, maternal periconceptional exposure to coffee, sweeteners and hairspray as well as maternal body mass index (BMI). Furthermore, we analyzed if the development of CDH might be influenced by chronic diseases like maternal hypothyroidism or maternal periconceptional folic acid intake, previous miscarriages or infertility treatment. We compared data of environmental risk factors obtained from 199 CDH patients born at the University Hospital of Bonn and the University Hospital of Mannheim between 2001 and 2019 to data of environmental risk factors obtained from 597 healthy control newborns born all over Germany in 2011.

## Methods

### Patients and families

We designed a case-control study with regard to CDH patients born at the University Hospitals of Bonn and Mannheim. Both hospitals represent the largest centers in Germany for the treatment and care of CDH newborns. The respective ethics committees of the University Hospital of Bonn and Mannheim approved our study. Prior to inclusion, written informed consent was obtained from all patients respectively their parents. For our analysis we used data of CDH newborns born in Bonn between 2001 and 2019 and born in Mannheim between 2001 and 2016. We did not contact families retrospectively in whom we knew, that the index patient had passed away. Initially, we contacted 647 CDH families. All families that consented to participate were re-contacted via phone for further explanations about the study’s design.

### Epidemiologic questionnaire

The families received our study questionnaire from CURE-Net, which is an ongoing, multicenter, population-based study initiated in 2009. The same questionnaire has been previously used to investigate environmental risk factors for urorectal malformations [15].

This questionnaire comprises of 14 pages including a standardized case report form to document the phenotype, family history and the presence of any co-morbid disorders. In addition, it surveys demographic information from patients and their families in accordance to the EUROCAT (European surveillance of congenital anomalies) report forms (http://www.eurocat-network.eu/). This information was amended for socio-demographic factors, the periconceptional and prenatal period, the child’s birth and the parents’ lifestyle regarding to nutrition or potential noxious agents.

### Study design

In detail, the following classification of risk factors were made for our study: Parental smoking as well as parental alcohol, coffee or sweetener intake and the use of hairspray was recorded both for the periconceptional time and the rest of pregnancy. Periconceptional was defined as 3 months before conception until the end of the first trimester. Maternal BMI was calculated using the height and weight at the beginning and end of pregnancy. Because CDH formation occurs within the first trimester as mentioned above, we focused on the abovementioned time frame for our analysis. Maternal chronic diseases were recorded as free text questions. Folic acid intake was documented with regards to date of initiation and dosage of supplementation. Previous miscarriages were listed and a previous infertility treatment was specified into two subgroups of hormonal treatment exclusively or followed by in vitro fertilization (IVF)/intracytoplasmic sperm injection (ICSI). Parental contact to animals was recorded by listing the respective animals*.*

### Response and control group

Of the 336 questionnaires sent out via mail, 201 were returned. We decided to exclude all CDH cases with chromosomal anomalies, suspected genetic syndromes or molecular diagnosis of a genetic syndrome. Associated malformations were recognized using the physician’s letters of the respective hospitalization. Finally, we compared data of 199 CDH cases born in Bonn and Mannheim between 2001 and 2019 with 597 infants without congenital malformations born in 2011. The control questionnaires were gathered just after births, the case questionnaires several years later. Since the CDH patients born in Bonn and Mannheim originally came from all over Germany to be delivered in these two centers, we used only healthy control newborns that were also collected from all over Germany. Their families received the same questionnaire directly after the child’s birth. The control group was not gender matched. To increase the power, we chose a 3:1 match control:cases. Controls with an identical distribution regarding the year of birth were not available.

### Statistical analysis

Nonparametric measurement methods (chi square-test, Mann-Whitney-U test) were used to calculate possible differences between cases and controls. For potential parental risk factors we calculated the odds ratio, 95% confidence interval (CI) and the *P*-value using binary logistic regression.

Explorative significance was defined by *P* < 0.05. Analyses were performed using the statistics software SPSS©, version 27 (International Business Machines Corporation (IBM), Armonk, NY).

## Results

Data of 199 CDH patients consisting of 85 female and 114 male patients born between 2001 and 2019 were compared with 597 healthy control infants (289 females and 305 males) born in 2011. Demographics of CDH and controls are shown in Table 1. The male-to-female ratio is hence 1.341 compared to 1.055 in the normal population, which corresponds to an odds ratio (OR) of 1.271 (95%-CI: 0.919, 1.757) for male gender (*P* = 0.163). 42 cases were treated at the University Hospital of Bonn and 157 of CDH cases were treated at the University Hospital of Mannheim. The majority of CDH-patients was born with a left-sided CDH (82.9%), 14.6% had a right-sided CDH and one patient presented with bilateral CDH. 66 cases (33.2%) required ECMO-assistance. 76.4% of the CDH-patients presented with isolated CDH defects and 21.6% were so-called “complex CDH-cases” with additional anomalies. Most of these associated defects were related to the cardiovascular system (62.8%) followed by malformations of the respiratory, genitourinary, central nervous or gastrointestinal system (Table 2). Both gestational age and birth weight of CDH cases were significantly lower compared with controls (*P* < 0.0001). There was no statistically significant difference between both groups according to the parent’s age at the time of child’s birth (*P* = 0.147 for maternal age and *P* = 0.502 for paternal age). Parent’s baseline characteristics according to both groups are shown in Table 3. Altogether we analyzed more than 25 different variables as potential risk factors. The impact of known risk factors for the development of CDH are summarized in Table 4 (univariate analyses) and Table 5 (multivariate analysis).

**Table 1:** Characteristics of CDH Patients and Control Group

	CDH patients (*n* = 199)	Control group (*n* = 597)	*P* value
Gender			0.146^c^
Male	114 (57.3%)	305 (51.1%)	
Female	85 (42.7%)	289 (48.4%)^a^	
Gestational age,	37	39	< 0.0001^d^
Median (range)	(32–41)	(21–45)^b^	
Birth weight,	3020 g	3350 g	< 0.0001^d^
Median (range)	(1600–4600)	(1400–6500)	
Twins	7 (3.5%)	11 (1.8%)	0.169^c^
Monozygotic	2 (28.6%)	3 (27.3%)	
Dizygotic	3 (42.9%)	8 (72.7%)	
Unknown	2 (28.6%)		

^a^Data was missing in 3 cases.

^b^Data was missing in 18 cases.

^c^Calculated by the Chi-square test.

^d^Calculated by the Mann-Whitney U-test

**Table 2:** Further Classification of CDH Patients

	CDH patients (*n* = 199)
Localisation^a^	
Left	165 (82.9%)
Right	29 (14.6%)
bilateral	1 (0.5%)
ECMO required^a^	
No	129 (64.8%)
Yes	66 (33.2%)
Associated malformations^a^	
None	152 (76.4%)
Yes	43 (21.6%)
Cardiovascular	34 (79.1%)
Genitourinary	3 (7%)
Centralnervous	2 (4.6%)
Gastrointestinal	1 (2.3%)
Other	3 (7%)

^a^Data was missing in 4 cases

**Table 3:** Parent’s Baseline Characteristics according to CDH Patients and Control Group

	CDH patients (*n* = 199)	control group (*n* = 597)	*P* value^c^
Maternal age, median (range)	32 (19–44)	31 (17–60)	0.147
Paternal age, median (range)	34 (20–56)	34 (19–72)^a^	0.502
Number of pregnancies, median (range)	2 (1–12)	2 (1–8)^b^	0.217
Maternal BMI before pregnancy, median (range)	23.2 (16.8–39.9)^a^	22.7 (16.4–56.1)^b^	0.526

*Abbreviation*: *BMI* body mass index (kg/m^2^)

^a^Data was missing in 6 cases

^b^Data was missing in 17 cases

^c^Calculated by Mann-Whitney U-test

**Table 4:** Univariate Associations between Maternal or Paternal Factors and CDH

	Control group (*n* = 597)	CDH patients (*n* = 199)	*P* value^a^
Maternal periconceptional smoking^b^			
No smoking	456 (76%)	161 (81%)	
Any smoking	133 (22%)	37 (19%)	0.265
1–5 cigarettes/day	38 (6.5%)	14 (7%)	0.896
6–10 cigarettes/day	36 (5.9%)	10 (5%)	0.515
> 10 cigarettes/day	59 (9.9%)	13 (6.5%)	0.153
Paternal periconceptional smoking^c^			
No smoking	385 (64%)	138 (69%)	
Any smoking	181 (31%)	54 (29%)	0.211
1–5 cigarettes/day	5 (0.8%)	9 (4.5%)	0.318
6–10 cigarettes/day	30 (5%)	14 (7%)	0.435
> 10 cigarettes/day	146 (24.5%)	31 (15.5%)	0.017
Maternal periconceptional alcohol intake^d^			
No	165 (28%)	55 (28%)	
Yes	266 (45%)	135 (68%)	**0.025**
Paternal periconceptional alcohol intake^e^			
No	81 (14%)	30 (15%)	
Yes	340 (57%)	156 (78%)	0.361
Maternal periconceptional coffee intake^f^			
No	99 (17%)	40 (20%)	
Yes	443 (74%)	155 (78%)	0.492
≥ 30 units/month	309 (52%)	128 (64%)	0.908
Maternal periconceptional sweetener intake^g^			
No	202 (34%)	101 (51%)	
Yes	163 (27%)	89 (45%)	0.624
≥ 30 units/month	50 (8%)	22 (11%)	0.481
Maternal periconceptional use of hairspray^h^			
No	470 (79%)	146 (73%)	
Yes	95 (16%)	49 (25%)	**0.011**
Maternal periconceptional BMI^j^			
< 18.5 kg/m^2^	23 (4%)	5 (2.5%)	0.376
18.5–24.9 kg/m^2^	390 (65%)	132 (66%)	0.764
25–29.9 kg/m^2^	87 (15%)	36 (18%)	0.229
≥ 30 kg/m^2^	74 (12%)	18 (9%)	0.202
Maternal hypothyreosis			
Yes	20 (3.3%)	13 (6.5%)	0.051
Maternal periconceptional folic acid intake			
No	385 (64%)	115 (58%)	
Yes	212 (36%)	84 (42%)	0.165
Previous miscarriages^k^			
No	438 (73%)	141 (71%)	
Yes	111 (19%)	53 (27%)	**0.04**
Pregnancy after infertility treatment^l^			
No	544 (91%)	177 (89%)	
Yes	35 (6%)	20 (10%)	0.052
Hormone treatment	11 (2%)	10 (5%)	**0.016**
Maternal periconceptional contact to animals^m^			
No	494 (83%)	160 (80%)	
Yes	73 (12%)	38 (19%)	**0.03**
Especially cats	40 (7%)	25 (13%)	**0.014**

^a^Calculated by the Chi-square test

^b^Data was missing in 9 cases in the control group and 1 case in the CDH group

^c^Data was missing in 31 cases in the control group and 7 cases in the CDH group

^d^Data was missing in 166 cases in the control group and 9 cases in the CDH group

^e^Data was missing in 176 cases in the control group and 13 cases in the CDH group

^f^Data was missing in 57 cases in the control group and 4 cases in the CDH group

^g^Data was missing in 232 cases in the control group and 9 cases in the CDH group

^h^Data was missing in 32 cases in the control group and 4 cases in the CDH group

^j^Data was missing in 23 cases in the control group and 8 cases in the CDH group

^k^Data was missing in 48 cases in the control group and 5 cases in the CDH group

^l^Data was missing in 18 cases in the control group and 2 cases in the CDH group

^m^Data was missing in 30 cases in the control group and 1 case in the CDH group

**Table 5:** Statistically Significant Risk Factors for CDH in Multivariate Analyses

	OR	95% CI	*P* value
Maternal periconceptional alcohol intake	1.639	1.101–2.440	0.015
Maternal periconceptional use of hairspray	2.072	1.330–3.229	0.001

*Abbreviations*: *OR* Odds Ratio, *CI* Confidence Interval

### Maternal exposure to tobacco, alcohol, coffee, sweeteners and hairspray

We could not find statistical significance for both maternal and paternal periconceptional smoking. The univariate analyses demonstrated statistical significance for maternal periconceptional alcohol intake (*P* = 0.025), which retained its significance using binary logistic regression (OR = 1.639; 95% CI: 1.101, 2.440, *P* = 0.015). There was no connection between the development of CDH and periconceptional maternal consumption of coffee or sweeteners. A significantly increased risk for CDH was observed with maternal periconceptional use of hairspray (OR = 2.072; 95% CI: 1.330, 3.229; *P* = 0.001).

### Maternal BMI and chronic diseases

Neither maternal underweight, nor maternal overweight or even obesity seemed to have an impact on the risk of CDH development, *P* -values for maternal BMI are shown in Table 4. Maternal hypothyroidism as chronic disease showed borderline significant association with CDH development (*P* = 0.051).

### Maternal periconceptional folic acid supplementation, previous miscarriages, and hormone substitution

There was no association between the development of CDH and periconceptional maternal folic acid intake (*P* = 0.165). Previous miscarriages and pregnancy as a result of hormone treatment showed association (*P* = 0.040 and 0.016) in univariate analyses but was not associated anymore when analyzed in a binary logistic regression model.

### Maternal periconceptional contact to animals

Maternal periconceptional contact to animals presented as significant (*P* = 0.03) in univariate analyses with an even lower *P* -value for maternal periconceptional contact to cats (*P* = 0.014), but both variables did not remain significant in multivariate analyses.

## Discussion

Here, in a case-control study we investigated potential environmental risk factors in 199 CDH patients compared to 597 healthy control newborns. Our analyses did not find association between parental periconceptional smoking and CDH. However, we observed significant association for maternal periconceptional alcohol intake. Accordingly, previous studies by Felix et al. in 2008 and McAteer et al. in 2014 described association between the occurrence of CDH and maternal periconceptional alcohol intake [11, 16]. In general, CDH is not part of the foetal alcohol related anomaly spectrum [17]. Nevertheless, previous studies report an association of maternal alcohol consumption and the expression of birth defects [18]. Yet, no pathophysiological concept exists that could causally link periconceptional or pregnancy related maternal alcohol consumption and the expression of structural birth defects. Likely, the observational association of maternal alcohol consumption with structural birth defects is more of an indirect association, warranting future studies, observational or experimental, to investigate this. The here described association between maternal periconceptional use of hairspray, the development of CDH, and the fact that hairsprays have already been discussed to possibly bear an impact on pregnancy and embryogenesis warrants further studies [19]. Especially, since a recent study by Karzi et al. in 2019 showed that the frequent use of hairspray and sunscreens is correlated with higher levels of parabens in hair of the pregnant women [20]. Recently, several experimental studies have investigated the embryonic toxicity of parabens using zebrafish larvae providing several lines of evidence, for the detrimental effects of parabens on zebrafish early-life stages with attention to its developmental toxicity [21, 22]. Observed features among zebrafish larvae where pericardial edema, yolk edema, blood stasis, reduction in blood circulation, reduced heartbeat and notochord curvature [23]. While parabens are commonly found in personal care products such as hair spray, it is unclear, whether the maternal use during the periconceptional period might exert toxic effects on human embryonic development. Hence, the current observation should warrant measurements of parabens in women using on a regular level hair spray or paraben containing care products and correlate the measured quantities in women to the toxic quantities used in the above mentioned zebrafish studies.

While previous studies suggested an association of periconceptional maternal overweight and CDH [24, 25] our study could not replicate this association (*P* = 0.526). The studies of García et al. published in 2016 [9] and Paoletti et al. published in 2020 [10] revealed a significant increased risk of bearing a child with CDH for mothers with an age ≥ 35 years (*P* < 0.001 and *P* = 0.0004). While our study did not replicate this association, median maternal age of both, case and control mothers in our study, was below 35 years of age. In 2014, McAteer et al. [11] described an association between maternal pregestational diabetes and CDH (*P* = 0.003). Our study could not replicate this finding (*P* = 0.827). Furthermore, we did not observe the previously described association between periconceptional maternal hypertension and CDH by Mesas Burgos et al. in 2019 [12]. In fact, we did not find any association between maternal chronic diseases of the cardiovascular or respiratory spectrum and CDH in their offspring. However, our study is suggestive (*P* = 0.051) for an association between maternal periconceptional hypothyreosis and CDH which should warrant further studies in the future.

Our study did not reveal any potential associations between maternal periconceptional intake of medical drugs and CDH. Here, Crider et al. had previously observed an association between periconceptional maternal intake of sulfonamides and CDH [26]. Furthermore, our results do not support maternal periconceptional folic acid supplementation to be protective in the prevention of CDH. However, prevention of CDH has not been an indication for maternal periconceptional folic acid supplementation.

Regarding periconceptional maternal vitamin supplementation, the respective questionnaire items were barely answered except for folic acid intake. This is most likely due to the fact, that in Germany, no monitoring or systematic information exists on what and how many vitamins should be supplemented during pregnancy, except for folic acid. Nevertheless, previous reports by Beurskens et al. in 2013 [27] describe an association between ‘dietary vitamin A intake during pregnancy below the recommended daily intake (800 μg per day) in normal-weight mothers’ and the risk of bearing a child with CDH (*P* = 0.01). Recently Carmichael et al. described low maternal qualitative diets and lack of riboflavin to be associated with CDH formation in the offspring [13]. Moreover, Turkmen et al. described lower maternal vitamin D (*P* = 0.019) and calcium levels (*P* < 0.001) to be associated with ‘pregnancies complicated by CDH’ and hypothesized that hypovitaminosis D may ‘play a vital role in the pathogenesis of CDH’ [28]. The exact mechanisms of different hypo- or hypervitamonsis and the expression of birth defects are vastly unknown. Yet, the observations made for transcription factor GATA-4, encoded by the *GATA4* gene (MIM #600576) and its function in a vitamin A-deficient embryonic environment could be of model character. *GATA4* belongs to the few genes identified to play a role in human CDH formation [29]. On the other hand, Feng et al. were able to show in an experimental mouse study, that the mouse embryos of vitamin A-deficient mothers, presented with a high incidence of cardiac defects [30]. Interestingly, they also found high methylation status in the CpG loci of *GATA4* gene with a low expression of GATA-4 mRNA from vitamin A-deficient group embryos. Hence, embryonic transcriptome maps of the developing diaphragm in vertebrates could yield further genetic drivers, that may play a role in (human) diaphragm development allowing for the identification of genetic drivers, that require certain vitamins or track elements. Here, hypomorphic alleles, possibly discovered by genome-wide association studies and a deficient intrauterine environment might lead to CDH or other common birth defects.

To the best of our knowledge, the present study is the first German study that assessed the potential association between CDH and various parental environmental risk factors. Here, we were able to recruit a large sample of CDH families and respective healthy controls newborns in a match of 1:3 (cases:controls) to yield maximal statistical power and to facilitate the detection of associations between potential risk factors and CDH.

Our study is limited by a potential recall bias among CDH case mothers or fathers due to the retrospective collection of data, which in some cases was carried out up to 17 years after the child’s birth whereas the control group received the questionnaire almost immediately after delivery. Furthermore, we cannot exclude, a potential bias in both groups, cases and controls, especially among case parents in that parents with an unhealthy lifestyle habit might not have been willing to participate due to guilt feelings. Nevertheless, our results suggest an increased direct or indirect risk for CDH formation to be associated with maternal periconceptional alcohol consumption regardless of the beverage chosen or the frequency of consumption. Furthermore, we observed an association between maternal periconceptional use of hairspray, which might point to an embryonic impact of parabens on embryonic development.

## Data Availability

“The datasets generated and analysed during the current study are not publicly available due to privacy of the participating families and sensitivity issues but are available from the corresponding author on reasonable request.

## Abbreviations

CDH: Congenital diaphragmatic hernia
ICSI: Intracytoplasmic sperm injection
IVF: In vitro fertilization
